# Supplementary material for: A Core-Shell Approach for Systematically Coarsening Nanoparticle–Membrane Interactions: Application to Silver Nanoparticles
Source: Nanomaterials (Basel). 2022 Nov 1;12(21):3859. doi: 10.3390/nano12213859 (PMC9656456; doi:10.3390/nano12213859)
Supplement: Supplementary file 1 [file nanomaterials-12-03859-s001.zip › nanomaterials-1969358-supplementary.pdf]

Supporting Information: A systematic core-shell approach for coarsening nanoparticle-membrane interactions: application to silver nanoparticles

Ankush Singhal* and G. J. Agur Sevink*

Leiden Institute of Chemistry, Leiden University, P.O. Box 9502, 2300 RA Leiden, The Netherlands

E-mail: a.singhal@lic.leidenuniv.nl; a.sevink@lic.leidenuniv.nl

Table S1: System composition with respect to NP size. The first column shows the membrane area of a considered lipid bilayer composed of DPPC, POPC, DOPC, and Chol in a 5:2:2:1 ratio. The second and third column shows the number of lipids and the number of water molecules and anti-freeze water molecules (WF) used for each nanoparticle.

Size (nm)	Membrane area (nm^2)	No. of lipids				No. of water molecule	
		DPPC	DOPC	POPC	Chol	W	WF
3	224.86	440	176	176	44	26054	1253
5	537.26	1024	408	408	204	45111	4472
10	2055.57	3840	1536	1536	768	330000	30000

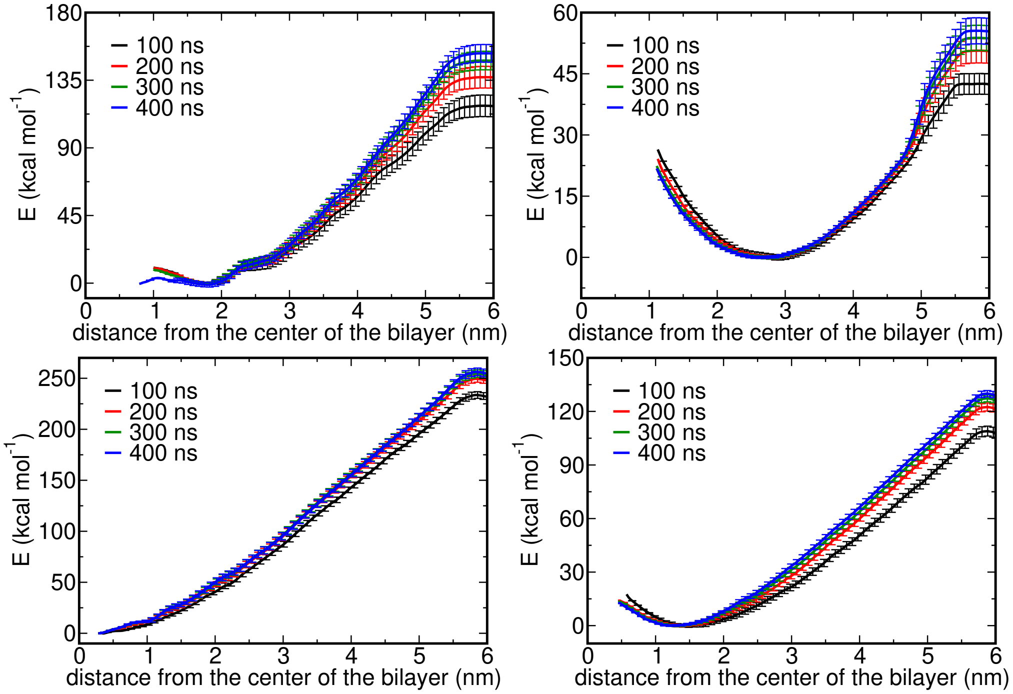

Figure S1: PMF showing convergence with time calculated for 3 nm (top) NP with C_{C1}-S_{C1} (top left) and C_{C3}-S_{C2} (top right) and 5 nm NP (bottom) for C_{C1}-S_{C1} (bottom left) and C_{C5}-S_{C2} (bottom right). The shaded region corresponds to the standard deviation calculated over 100 iteration steps using the bootstrapping technique implemented in GROMACS.

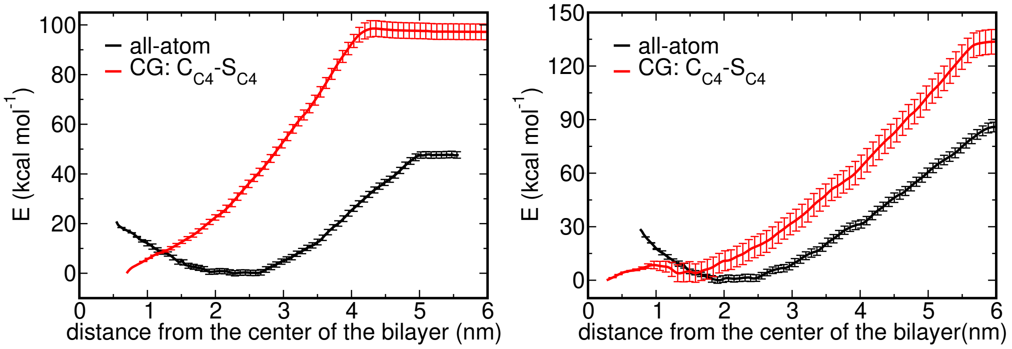

Figure S2: PMFs calculated for the insertion of NP parametrized as a single CG bead for sizes 3 nm (left) and 5 nm (right) in the lipid bilayer composed of DPPC, DOPC, POPC, and CHOL in a 5:2:2:1 ratio. The shaded region corresponds to the standard deviation calculated over 100 iteration steps using the bootstrapping technique implemented in GROMACS.

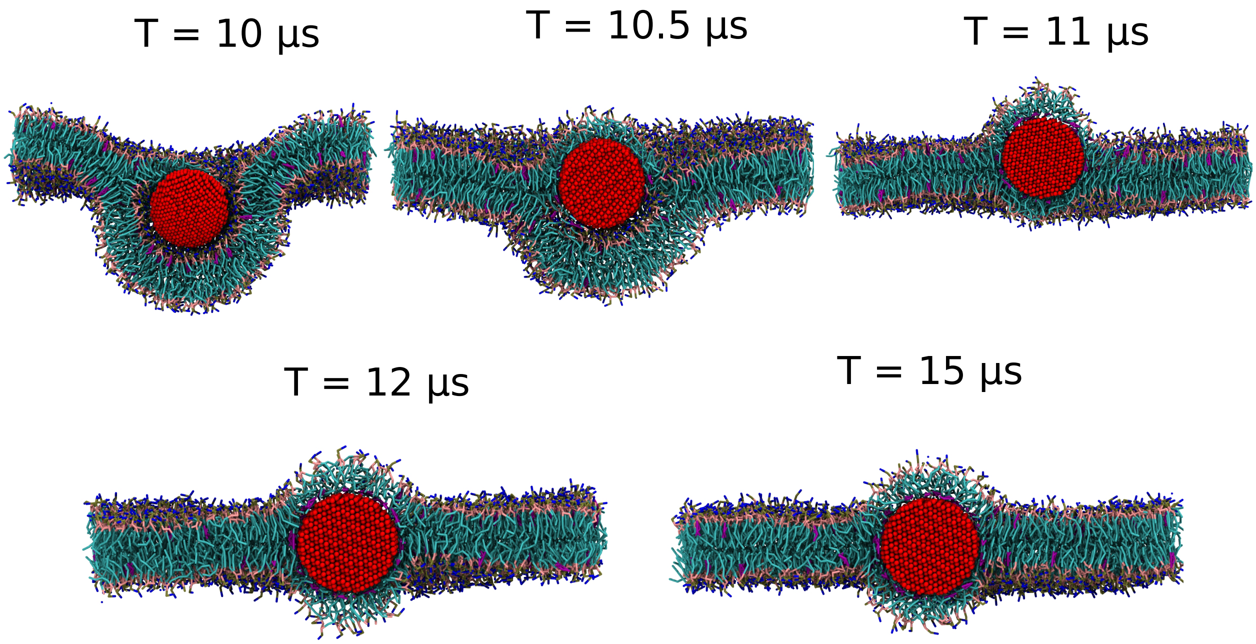

Figure S3: Simulation snapshots of an extended simulation showing the evolution of 5 nm NP interacting with a lipid membrane composed of DPPC, DOPC, POPC, and CHOL in a 5:2:2:1 ratio. The snapshot corresponds to C_{C2}-S_{C2} representation. As the nature of the lipids is not crucial at the stage, all the lipids are shown as blue and cholesterol as pink sticks, while each NP CG bead is represented as a red sphere.

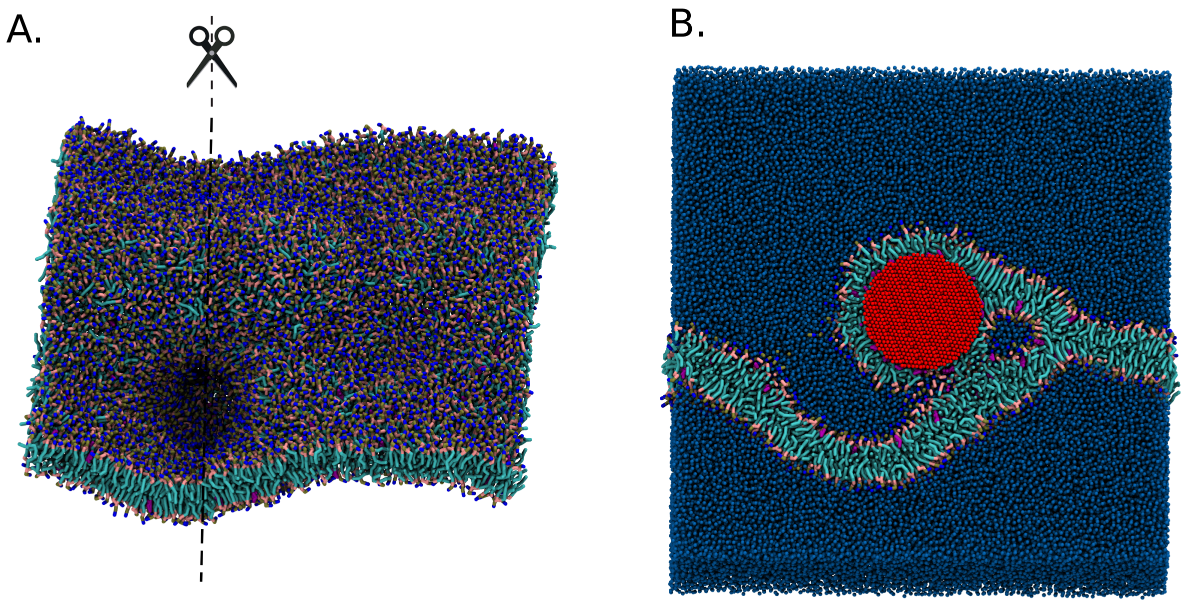

Figure S4: Simulation snapshots showing 10 nm Ag NP interacting with a lipid membrane composed of DPPC, DOPC, POPC, and CHOL in a 5:2:2:1 ratio. (A) The front view of the simulation snapshot showing membrane undulation induced by Ag NP insertion. (B) The snapshot produced by slicing (A) in the x-direction shows the formation of a three-way junction and a water channel. As the nature of the lipids is not crucial at the stage, all the lipids are shown as blue and cholesterol as pink sticks, while each NP CG bead is represented as a red sphere. The water in the second snapshot is shown as a blue coloured sphere.
